# Machine learning reveals microbiome differences by periodontitis severity

**DOI:** 10.1371/journal.pone.0349686

**Published:** 2026-05-21

**Authors:** Soo Hyun Seo, Jae Won Lee, Sujin Oh, Jin-Sil Hong, Ban Seok Lee, Sun Jae Kwon, Keun-Suh Kim, Jung Soo Park, Ju Sun Heo, Ki Hoon Ahn, Hyo‐Jung Lee, Kyoung Un Park

**Affiliations:** 1 Department of Laboratory Medicine, Seoul National University Bundang Hospital, Seongnam, Republic of Korea; 2 Department of Laboratory Medicine, Seoul National University College of Medicine, Seoul, Republic of Korea; 3 Department of Genomic Medicine, Seoul National University Bundang Hospital, Seongnam, Republic of Korea; 4 Accugene Inc., Incheon, Republic of Korea; 5 Department of Periodontology, Section of Dentistry, Seoul National University Bundang Hospital, Seongnam, Republic of Korea; 6 Department of Periodontology, Korea University Anam Hospital, Seoul, Republic of Korea; 7 Department of Pediatrics, Seoul National University Children’s Hospital, Seoul, Republic of Korea; 8 Department of Pediatrics, Seoul National University College of Medicine, Seoul, Republic of Korea; 9 Department of Obstetrics and Gynecology, Korea University College of Medicine, Seoul, Republic of Korea; University of Pennsylvania, UNITED STATES OF AMERICA

## Abstract

Periodontitis is a chronic inflammatory disease driven by microbial dysbiosis, yet the microbial signatures associated with severity remain incompletely understood. This study investigated changes in subgingival microbial composition across clinically defined severity groups and evaluated the performance of microbiome-based machine-learning models for classifying periodontitis severity. Subgingival plaque samples from 84 patients were analyzed using 16S rRNA gene sequencing. Microbial diversity showed a modest decreasing trend with increasing severity, although differences were not statistically significant. Five machine learning models were applied to classify periodontitis. Random Forest and XGBoost achieved AUC values of 0.98, indicating statistically significant classification performance (p < 0.05) after feature selection. Validation using three external cohorts demonstrated substantial performance variability across populations, reflecting differences in oral microbiome composition, sample type, and periodontal status definitions. Feature importance analysis identified *Fusobacterium*, *Campylobacter*, *Stomatobaculum*, *Leptotrichia* and *Segatella* as key contributors to periodontitis severity classification, consistent with their established roles in periodontal dysbiosis. These findings highlight the potential of microbiome-based models for classifying periodontitis severity while underscoring the need to incorporate diverse populations and robust feature-selection strategies to enhance generalizability.

## Introduction

Periodontitis, a chronic inflammatory disease that affects the supporting structures of the teeth, leads to progressive bone loss and potential tooth loss if left untreated. It is one of the most prevalent oral diseases worldwide and is strongly associated with microbial dysbiosis, wherein opportunistic pathogens outcompete benign commensal species and drive disease progression [1]. Periodontitis severity is clinically assessed using standardized criteria based on clinical attachment loss (CAL), probing depth (PD), radiographic bone loss, and the extent of tooth loss, as outlined in the 2018 AAP/EFP classification [2]. Severity is typically categorized as mild (Stage I), moderate (Stage II), or severe (Stage III–IV) depending on the extent and complexity of tissue destruction. These parameters guide treatment strategies, ranging from non-surgical mechanical debridement in mild cases to surgical intervention and regenerative therapy in advanced disease. Accurate assessment of severity is therefore essential not only for diagnosis but also for determining prognosis and guiding clinical decision-making.

Several microbial taxa have been consistently associated with increasing periodontitis severity across previous studies. The best-characterized markers include members of the red complex—*Porphyromonas gingivalis, Tannerella forsythia*, and *Treponema denticola*—which are strongly linked to deep periodontal pockets and advanced tissue destruction [3]. Orange complex taxa such as *Fusobacterium, Campylobacter,* and the recently reclassified *Segatella* (formerly *Prevotella*) have been reported to increase progressively with disease severity and are considered important bridging species that facilitate the establishment of late-stage pathogens. In addition, green complex bacteria play a crucial role in the initiation and progression of periodontitis, with specific species, such as *Capnocytophaga* species, notably associated with the severity of periodontal disease [4].

The human oral microbiome plays a crucial role in maintaining periodontal health and alterations in its composition are key contributors to the development of periodontitis. Understanding these microbial changes is essential for early diagnosis and effective treatment. Recent advances in high-throughput sequencing technologies have enabled the comprehensive characterization of the oral microbiome [5]. Previous studies have shown that early microbial and host immune shifts occur even at the asymptomatic gingival stage [6], and cross-cohort metagenomic analyses have identified universal microbial signatures for periodontitis [7]. In addition, machine-learning models using oral microbiome data, such as salivary pathogen-based classifiers, have demonstrated high accuracy in distinguishing periodontitis cases from controls [8]. However, despite these advances, most prior work has focused either on experimental gingivitis models, biomarker discovery without severity stratification, or limited pathogen panels.

In this study, we aimed to investigate the relationship between microbial diversity and periodontitis severity, while evaluating the performance of machine learning models in classifying periodontitis based on microbiome data. We assessed the changes in microbial composition across groups with differing disease severities and applied various machine-learning algorithms to develop predictive models for classifying periodontitis severity. To assess their clinical applicability, we explored the generalizability of these models across different populations. By integrating microbiome profiling with machine learning, this study aimed to advance our understanding of the microbial factors associated with periodontitis and contribute to the development of more accurate diagnostic approaches. This model could enable the creation of a more efficient tool for assessing periodontitis severity, ultimately contributing to improved clinical decision-making and personalized treatment strategies.

## Materials and methods

### Sample collection

This study was designed as a retrospective observational study. Subgingival plaque samples and corresponding clinical data were previously collected during routine periodontal examinations at the Department of Periodontology, Seoul National University Bundang Hospital. A total of 84 patients diagnosed with periodontitis participated in the study (45 males and 39 females; mean age, 44.5 years). All periodontal probing depths were recorded.

For analytical purposes, periodontitis severity was classified at the patient level based on the deepest periodontal pocket depth (PPD) recorded per individual. Specifically, patients were categorized as follows: mild = PPD ≤ 3 mm; moderate = PPD > 3 mm and < 6 mm; and severe = PPD ≥ 6 mm, as deeper periodontal pocket depths were considered to more reliably reflect the subgingival microbiome profile [9]. If a patient presented with at least one site meeting the threshold, the highest corresponding severity was assigned [10,11]. Participants classified in the mild group (PPD ≤ 3 mm) were considered periodontally healthy for the purposes of this study, as this threshold reflects the absence of clinical periodontal breakdown. Subgingival plaque samples were collected from the two deepest periodontal pockets, preferentially selecting teeth in opposite quadrants. When multiple teeth exhibited similar probing depths, sampling was guided by the WHO CPITN criteria, prioritizing teeth #16, #26, #36, and #46 [12]. The deepest measurement among the six standard periodontal sites per tooth was used to determine the sampling location. Prior to sampling, debris was removed with gauze, and supragingival plaque was carefully eliminated using a curette. Subgingival plaque was then collected with minimal contact to the tooth surface. To avoid the potential influence of bleeding induced during probing, the sampling and probing procedures were performed at least one day apart. To minimize contamination, all sampling instruments were plasma-sterilized, and plaque collection was performed under gloved, contact-free conditions. All samples were stored at −80 °C until analysis.

Patients were excluded if they had used steroids, immunosuppressants, rheumatic medications, or antibiotics within the past month. The collection of subgingival plaque samples and clinical data was approved by the Institutional Review Board of Seoul National University Bundang Hospital (IRB no. B-1511-322-303), and written informed consent was obtained from all participants at the time of sample collection. The secondary analysis of archived samples and clinical records for the present study was subsequently approved by the Institutional Review Board of Seoul National University Bundang Hospital (IRB no. B-2406-906-301). Clinical data and archived subgingival plaque samples were accessed for research purposes after Institutional Review Board approval on 19/02/2025. The authors had limited access to identifiable patient information during data extraction, and all data were anonymized prior to analysis.

### 16S rRNA gene sequencing

We performed 16S rRNA gene sequencing as previously described [13]. DNA was extracted from all samples using the QIAamp DNA Microbiome Kit (QIAGEN, Venlo, Netherlands) according to standard protocols. DNA quality was assessed using the Qubit dsDNA HS Assay Kit (Thermo Fisher Scientific Inc., Waltham, MA, USA). Polymerase chain reaction (PCR) targeting the V3–V4 hypervariable regions of the 16S rRNA genes was conducted using the KAPA HiFi HotStart ReadyMix PCR kit (Roche, Basel, Switzerland). The primers used for PCR amplification were 519F: 5′-CCT ACG GGNGGC WGC AG-3′ and 806R: 5′-GAC TAC HVGGG TAT CTA ATC C-3′, generating amplicons of approximately 290 bp. Libraries were constructed using the Nextera XT DNA Library Preparation kit (Illumina, San Diego, CA, USA) and pooled to achieve a final loading concentration of 8 pM. Paired-end sequencing (2 × 300 bp) was performed using the Illumina MiSeq platform.

### Data processing and statistical evaluation

For downstream analysis, 16S rRNA sequences were processed using Quantitative Insights into Microbial Ecology 2 (QIIME2; version 2024.5) [14]. Sequence filtering was performed using the Divisive Amplicon Denoising Algorithm 2 (DADA2) [15], and amplicon sequence variants (ASVs) were taxonomically classified using the SILVA database (version 138.2) [16]. To assess microbiome richness and evenness, two diversity indices were calculated. Alpha diversity was measured using Shannon and observed feature indices on the QIIME2 platform. The Mann–Whitney–Wilcoxon test was used to identify statistically significant differences in diversity indices. Beta diversity, which reflects differences in microbial community composition among samples, was calculated using the Bray–Curtis distance and Jaccard dissimilarity metrics. Principal coordinate analysis (PCoA) was performed based on these metrics, and the results were visualized in two-dimensional plots. Permutational multivariate analysis of variance (PERMANOVA) was applied to test for statistically significant differences in beta diversity. Statistical significance was set at p ≤ 0.05.

### Development of machine learning model for classification of periodontitis severity

A microbiome-based severity classification model was constructed to differentiate the severity of periodontitis based on genus-level microbial profiles. Five different machine learning (ML) algorithms were evaluated: Support Vector Machine (SVM), k-nearest neighbors (KNN), Decision Tree, Random Forest (RF), and extreme gradient boosting (XGBoost, www.xgboost.ai). To assess the predictive performances of these algorithms, we applied four-fold cross-validation and fine-tuned the models through hyperparameter optimization using Grid Search [17]. To further enhance model performance, feature selection was carried out using the Mann-Whitney test to identify statistically significant genera for inclusion in the final models. No class imbalance correction methods, such as oversampling, undersampling, or class weighting, were applied during model training, as the class distribution was relatively balanced across severity groups.

### Validation datasets

The final XGBoost model was validated using three independent datasets from different populations: Chinese (Hong Kong; PRJNA477241) [18], Korean (Korea University) [19], and Spanish/Portuguese (PRJNA774299 and PRJNA774981) [20]. The validation Bioprojects were selected from publicly available 16S rRNA sequencing datasets based on the following criteria: (1) inclusion of periodontitis and periodontally healthy subjects, (2) availability of clearly defined oral sample types relevant to our study design, and (3) availability of sufficient metadata to allow phenotype-based grouping and downstream validation. Although all validation cohorts used V3–V4 16S sequencing and were therefore broadly comparable to our dataset, they differed in both sampling sites and periodontal status definitions. The Chinese and Korean cohorts collected subgingival plaque samples similar to ours, whereas the Spanish/Portuguese cohort used non-plaque oral samples, which differ substantially in microbial composition. Each dataset was processed using the same bioinformatics pipeline to generate normalized genus-level abundance tables. The selected features were applied to each validation dataset. The predictive performance of each of the ML models was evaluated using multiple metrics, including the area under the receiver operating characteristic curve (AUC), accuracy, F1 score, sensitivity, and specificity.

## Results

### Microbial diversity analysis

Of the 84 samples, 25 were classified as mild periodontitis, 37 as moderate, and 22 as severe. In the comparison of oral conditions among the mild, moderate, and severe periodontitis groups, significant intergroup differences were observed in Missing, Retained Natural Tooth, and Implant variables. Post hoc analysis using the Bonferroni correction revealed that these differences were statistically significant only between the mild and severe groups (S1 and S2 Tables).

**Fig 1:**
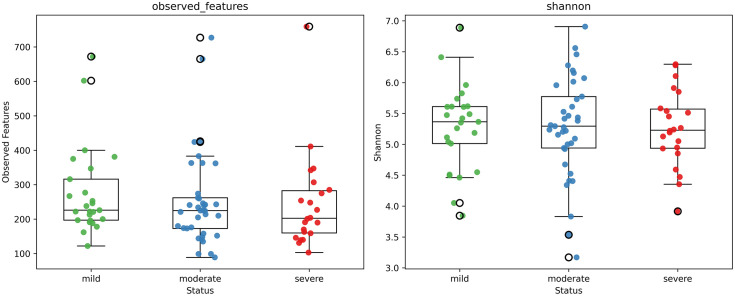
Diversity analysis results - α-diversity analysis. The Shannon index and observed feature index were used to assess species diversity. A slight decreasing trend in diversity was observed with increasing disease severity, but the difference was not statistically significant.

**Fig 2:**
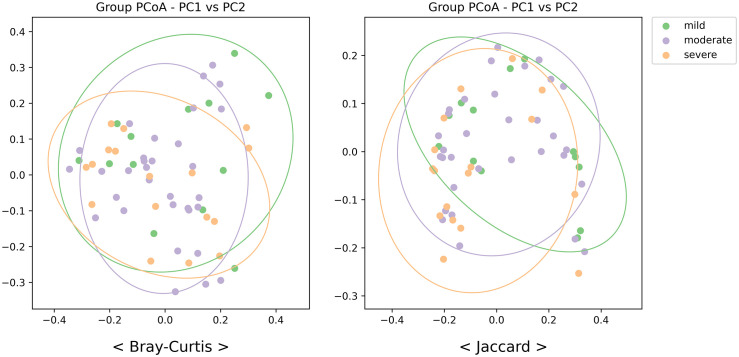
Diversity analysis results - β-diversity analysis. Principal coordinate analysis (PCoA) was performed based on Bray-Curtis distance and Jaccard distance. PERMANOVA revealed no significant separation among the groups based on Bray–Curtis dissimilarity (p = 0.39), whereas Jaccard distance showed a significant difference among the groups (p = 0.02). PCoA: principle coordinate analysis; PC1: Principal Coordinate 1; PC2: Principal Coordinate 2.

Before applying machine learning, a comprehensive microbiome analysis was conducted to investigate the differences in microbial diversity among the mild, moderate, and severe periodontitis groups and to better understand the microbial characteristics associated with disease progression. The Shannon index and observed features were used to evaluate microbial richness and evenness across the three groups. Pairwise Wilcoxon rank-sum tests between the mild and severe periodontitis groups showed no statistically significant differences in alpha diversity (Shannon index, p = 0.58; observed features, p = 0.65), although a decreasing trend in species diversity was observed with increasing disease severity (Fig 1). To assess overall differences in microbial community composition, beta diversity was analyzed using Bray–Curtis and Jaccard distance metrics (Fig 2). PERMANOVA revealed no significant separation among the groups based on Bray–Curtis dissimilarity (p = 0.39), whereas Jaccard distance showed a significant difference among the groups (p = 0.02). Despite the considerable overlap observed in the PCoA plots, the Jaccard-based result indicates that differences among the three groups may be partly driven by taxon membership, while abundance-related changes appear more subtle.

### Machine learning performance

Using the microbiome data from these samples, machine learning models were developed to classify periodontitis severity. Five different models—SVM, KNN, Decision Tree, RF, and XGBoost—were evaluated using two approaches. The first utilized all microbiome data, whereas the second applied feature selection using the Mann–Whitney U test to identify microbial taxa that differed significantly between mild and severe periodontitis samples. Feature selection was performed using the Mann–Whitney U test, identifying 15 microbial taxa (*Fusobacterium*, *Campylobacter*, *Stomatobaculum*, *Leptotrichia*, *Segatella* (formerly *Prevotella*), *Olsenella*, *Lautropia*, *Megasphaera*, *Sphingomonas*, *Phocaeicola*, *Escherichia-Shigella*, *Alloscardovia*, *Cutibacterium, Howardella,* and *Cupriavidus*) with statistically significant differences between mild and severe cases. After feature selection, model performance substantially improved, with AUC values increasing to 0.69 for SVM, 0.50 for KNN, 0.71 for Decision Tree, 0.98 for RF, and 0.98 for XGBoost. Among these, RF and XGBoost demonstrated the highest predictive capability. The RF model achieved an AUC of 0.98, sensitivity of 0.86, specificity of 1.00, and accuracy of 0.93, with XGBoost showing comparable performance (S3 Table).

### Model validation on independent datasets

**Fig 3:**
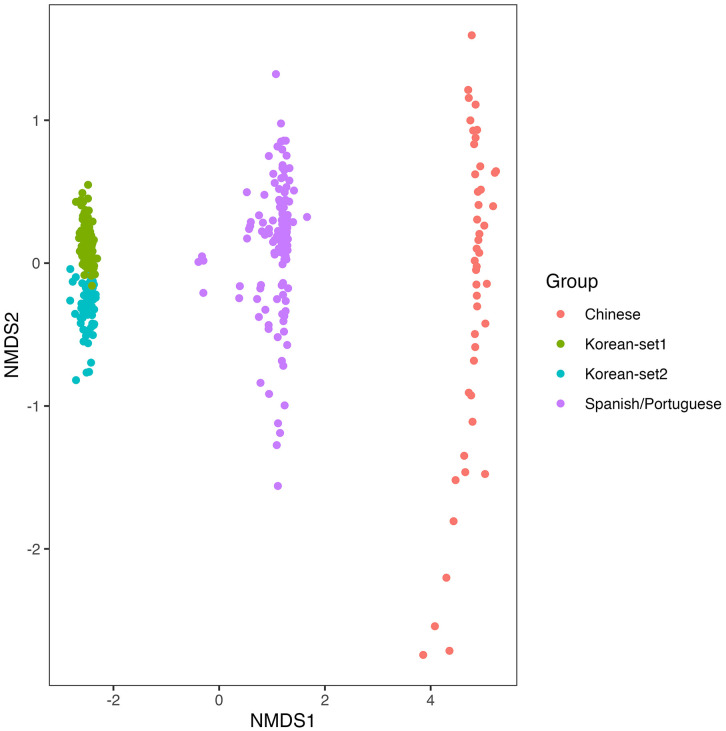
NMDS plot based on Bray–Curtis dissimilarity comparing oral microbiome compositions among Korean-set1, Korean-set2, Chinese (SRA Project: PRJNA477241), and Spanish/Portuguese (SRA Projects: PRJNA774981 and PRJNA774299) cohorts. Although cohort-level clustering patterns were visually observed, PERMANOVA did not identify statistically significant differences based on Bray–Curtis dissimilarity. NMDS: Non-metric Multidimensional Scaling.

**Fig 4:**
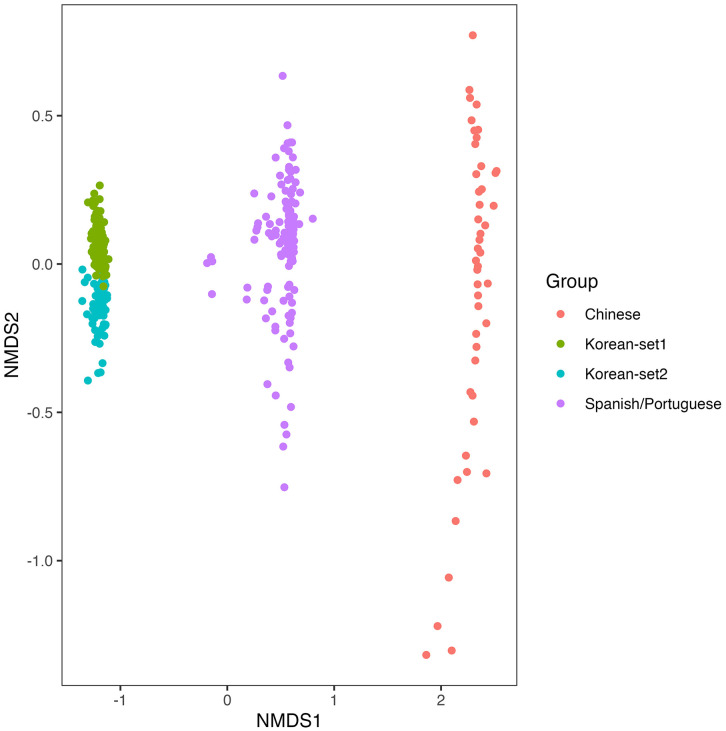
NMDS plot based on Jaccard distance comparing oral microbiome compositions among Korean-set1, Korean-set2, Chinese (SRA Project: PRJNA477241), and Spanish/Portuguese (SRA Projects: PRJNA774981 and PRJNA774299) cohorts. PERMANOVA identified significant differences among cohorts based on Jaccard distance, suggesting variation in taxon membership across populations. NMDS: Non-metric Multidimensional Scaling.

To validate the performance of the XGBoost model, independent validation datasets from Chinese (Hong Kong; PRJNA477241; mild, n = 21; severe, n = 25), Spanish/Portuguese (PRJNA774299; mild, n = 21; severe, n = 35; PRJNA774981; mild, n = 28; severe, n = 37), and Korean (Korea University; mild, n = 26; severe, n = 30) populations were used. The validation results exhibited notable performance variation across ethnic groups. However, because the validation cohorts differed in sample type and periodontal status classification criteria, direct comparisons across studies are limited. While the model performed well on the Korean validation dataset (sensitivity, 0.67; specificity, 0.90; accuracy, 0.85), its performance on the Chinese and Spanish/Portuguese datasets was notably lower, with reduced specificity and accuracy (S4 Table). To investigate the potential sources of these discrepancies, non-metric MDS analyses were performed using both Bray–Curtis and Jaccard distance metrics. As shown in Figs 3 and 4, cohort-level separation was observed among different ethnic groups, indicating marked differences in oral microbiome composition. PERMANOVA confirmed that these differences were statistically significant, suggesting that microbial profiles vary by population and may limit the generalizability of the model. Class-level composition bar charts sorted by severity and cohort, along with mean profiles, have been provided as S1 Fig.

### Key microbial taxa identified by the model

**Fig 5:**
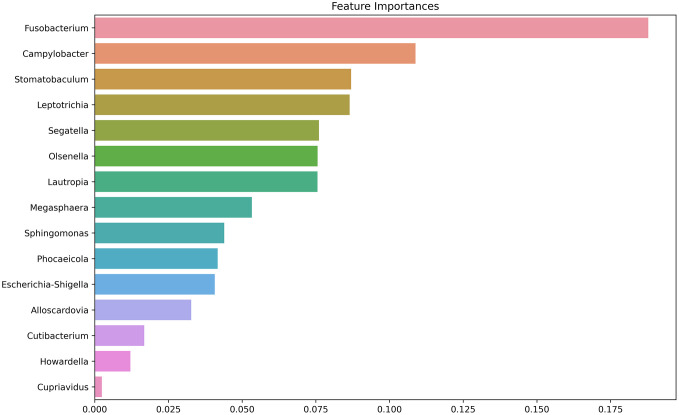
The bar chart displays the relative importance of microbial genera in the predictive model. *Fusobacterium* shows the highest importance, followed by *Campylobacter* and *Stomatobaculum*, suggesting their strong association with periodontitis.

To better understand the microbial contributors to periodontitis classification, we analyzed feature importance scores derived from the XGBoost model. Fig 5 presents the ranking of microbial taxa based on their importance in the model’s decision-making process. The analysis revealed that *Fusobacterium* had the highest feature importance score, indicating it was the most influential taxon for classification. Other key taxa included *Campylobacter*, *Stomatobaculum*, and *Leptotrichia*, which also demonstrated high importance, suggesting their relevance in distinguishing between severe and mild periodontitis. Additional taxa such as *Segatella*, *Olsenella*, and *Lautropia* contributed significantly to model performance, while *Megasphaera*, *Sphingomonas*, and *Escherichia-Shigella* were moderately important. We compared the mean relative abundance of the 15 selected microbial taxa across the three severity groups (mild, moderate, and severe). *Fusobacterium*, *Segatella*, *Leptotrichia*, *Campylobacter*, *Lautropia*, *Megasphaera*, *Stomatobaculum*, *Alloscardovia*, and *Phocaeicola* showed the highest abundance in the severe group. *Escherichia*–*Shigella*, *Olsenella*, *Sphingomonas*, and *Cupriavidus* were more abundant in the mild group, while *Cutibacterium* and *Howardella* showed slightly higher abundance in the moderate group. It is possible that *Escherichia*-*Shigella*, *Alloscardovia*, *Cutibacterium,* and *Cupriavidus* represent contaminants due to their known presence in the external environment or as skin commensals; however, a formal contamination check was not performed.

## Discussion

In this study, we investigated the relationship between microbial diversity and periodontitis severity and evaluated the performance of machine learning models trained on microbiome data. Although microbial diversity showed a decreasing trend with increasing disease severity, the differences were not statistically significant. This pattern suggests that periodontitis progression is accompanied by gradual shifts in community structure rather than abrupt changes in overall diversity. The relationship between microbial diversity and periodontitis remains controversial, with previous studies reporting conflicting findings—some indicating reduced diversity, others increased, and some showing no clear difference. These inconsistencies likely reflect differences in study populations, sampling sites, and analytical methodologies.

Feature importance analysis identified *Fusobacterium*, *Campylobacter*, and *Segatella* (formerly Prevotella), prominent members of Socransky’s orange complex [21], as the most influential taxa for periodontitis classification. Their enrichment is consistent with their established roles in disease pathogenesis, acting as bridging species that facilitate the colonization of late-stage red-complex pathogens. *Fusobacterium*, a Gram-negative anaerobe, has been consistently reported as a keystone species in periodontal dysbiosis, promoting biofilm maturation and eliciting inflammatory responses that drive tissue destruction [22,23]. Likewise, *Campylobacter* species show increased transcriptional activity in periodontitis, particularly within subgingival plaques, reflecting adaptation to the altered anaerobic and proteolytic microenvironment [24]. *Segatella*, recently reclassified from *Prevotella*, includes species such as *S. intermedia* and *S. melaninogenica*, which are frequently enriched during the transition from health to moderate–severe disease [25]. *Stomatobaculum*, previously reported to be abundant in both periodontal pockets and root canal infections, was likewise elevated in our dataset, suggesting a broader role across oral disease contexts [26].

The marked improvement in model performance when using feature-selected taxa compared to the full dataset underscores the importance of identifying biologically relevant microbial markers to enhance predictive accuracy. Although the canonical red-complex bacteria (*Porphyromonas gingivalis*, *Tannerella forsythia*, and *Treponema denticola*) were not among the most discriminative features, the predominance of orange-complex taxa supports the view that disease severity is accompanied by a gradual ecological shift rather than abrupt colonization by late pathogens.

Several taxa identified as key contributors to severity classification, including *Fusobacterium*, have been reported as part of the core oral microbiome present across health, gingivitis, and periodontitis [27,28]. These taxa are widely distributed in subgingival plaque but tend to increase in abundance with disease progression [27]. Importantly, previous microbiome indices have emphasized microbial imbalance rather than the presence or absence of specific taxa, often using ratios of disease-associated to health-associated taxa to quantify dysbiosis [28–30]. In this context, our findings suggest that core microbiome taxa can contribute to severity classification through their relative enrichment patterns. Because our study focuses on discriminating disease severity rather than binary health–disease status, these taxa may provide informative signals by capturing gradual ecological shifts across disease stages.

The observed enrichment of *Fusobacterium* and *Campylobacter* with increasing periodontitis severity may reflect microbial adaptation to the progressively anaerobic and inflammatory environment of deep periodontal pockets. These taxa initiate inflammation and contribute to tissue destruction by evading host immunity, potentially creating an environment conducive to the colonization and synergistic activity of other pathogenic bacteria, including the Red Complex, thereby accelerating disease severity. Nevertheless, because this study is cross-sectional, the observed associations represent correlations rather than evidence of temporal microbial transitions or disease progression. Although previously proposed microbiome indices have demonstrated the ability to capture longitudinal changes following periodontal treatment [31,32], our dataset did not include post-treatment follow-up samples and therefore could not address this question directly. In the present study, the mild group functioned as an operational periodontally healthy reference within a severity-based framework. Future prospective longitudinal studies incorporating treatment response data will be important to evaluate whether the microbial patterns identified here also have prognostic or monitoring value.

Finally, cross-population validation revealed marked variation in model performance across ethnic groups, highlighting potential influences of population-specific oral microbiome profiles. Differences in sampling procedures, sequencing pipelines, and case definitions may have contributed to the observed variability, indicating that these differences are likely multifactorial rather than attributable solely to ethnic variation. These findings highlight the importance of incorporating diverse cohorts and accounting for both biological and methodological heterogeneity to improve model generalizability and robustness.

Despite providing valuable insights, this study has several limitations. First, the relatively small sample size may restrict the generalizability of the findings. Second, potential confounding factors—such as smoking, systemic diseases, medication use, age, and oral hygiene status—may also have influenced the observed associations. These variables were not incorporated into the machine learning model, and their absence may introduce residual confounding or bias in the observed microbiome–severity relationships. Third, the absence of an independently recruited periodontally healthy control group may limit broader interpretability, as the mild group was used as the operational healthy reference in this study. Additionally, the exclusion of individuals with moderate periodontitis may have limited our ability to capture microbial transitions occurring during intermediate disease stages. Last, we did not include contamination control, which limits our ability to fully rule out the presence of low-abundance contaminants, although the high biomass of periodontal pocket samples reduces this likelihood.

Although not addressed in the present study, these aspects warrant investigation in future prospective studies. Periodontitis severity in this study was classified based on the deepest PPD rather than the 2018 AAP/EFP staging and grading system. While the 2018 framework is the clinical standard, our retrospective analysis was constrained by the absence of longitudinal radiographic and systemic data required for precise staging. Furthermore, we candidly acknowledge that relying on a single-site PPD without inflammatory metrics (e.g., bleeding on probing) may limit the biological interpretation of our findings, as it may reflect the physical ‘niche’ rather than the complete pathological state of the patient. Nevertheless, given our focus on the relationship between microbial habitat and dysbiosis, PPD served as a reproducible and biologically relevant parameter [9,10,33]. Future prospective studies incorporating full clinical staging and inflammatory markers will be essential for a more holistic model.

In addition, the variability observed across validation cohorts highlights the need for multi-cohort training frameworks that account for population-level differences in oral microbiome composition. Although expanding validation cohorts could help contextualize potential batch effects, identifying additional cohorts with comparable oral sample types and clearly defined periodontitis severity information remains challenging. Consequently, future multi-center studies employing standardized sampling protocols and harmonized analytical workflows will be essential to address this limitation and improve generalizability. Advances in artificial intelligence, including deep learning approaches, may further improve the modeling of complex microbial interactions beyond what is achievable with conventional feature selection methods. Future studies incorporating multi-omic datasets and geographically diverse populations will be essential for developing robust, generalizable, and clinically applicable models for periodontitis severity classification.

## Supporting information

S1 Table Participant demographic and clinical characteristics. (DOCX)

S2 Table Tooth-level clinical measurements and periodontal parameters. (DOCX)

S3 Table Comparison of model performance across classification algorithms. (DOCX)

S4 Table Performance metrics of XGBoost model according to populations. (DOCX)

S1 Fig Class-level microbial composition by severity in individual cohorts (A) and in the combined dataset (B). (TIF)
