# Supplementary material for: Machine learning reveals microbiome differences by periodontitis severity
Source: PLoS One. 2026 May 21;21(5):e0349686. doi: 10.1371/journal.pone.0349686 (PMC13193413; doi:10.1371/journal.pone.0349686)
Supplement: S1 Table — (DOCX) [file pone.0349686.s001.docx]

S1 Table. Participant demographic and clinical characteristics.

| Variable |  | mild | moderate | severe | Total | P-value |
| --- | --- | --- | --- | --- | --- | --- |
| N |  | 25 | 37 | 22 | 84 |  |
| Age (yr, Mean ± SD) |  | 36.4±9.4 | 42.2±14.5 | 57.5±12.1 | 44.5±14.9 | p < 0.001 |
| Height (cm, Mean ± SD) |  | 166.3±11.0 | 167.6±9.0 | 167.5±10.0 | 167.1±9.8 | 0.876 |
| Weight (Kg, Mean ± SD) |  | 69.7±28.2 | 78.2±27.7 | 69.7±11.9 | 73.4±25.2 | 0.383 |
| BMI (kg/m2, Mean ± SD) |  | 24.7±6.6 | 27.4±7.9 | 24.7±2.8 | 25.9±6.6 | 0.258 |
| Decay (N, Mean ± SD) |  | 0.2±0.8 | 0.1±0.6 | 0.1±0.3 | 0.2±0.6 | 0.687 |
| Missing (N, Mean ± SD) |  | 0.6±1.4 | 1.2±2.6 | 3.0±2.9 | 1.5±2.6 | 0.002 |
| Filling (N, Mean ± SD) |  | 3.7±3.7 | 5.5±4.8 | 6.2±5.5 | 5.2±4.7 | 0.169 |
| Implant (N, Mean ± SD) |  | 0.1±0.4 | 0.5±1.8 | 1.4±2.2 | 0.6±1.7 | 0.037 |
| Pontic (N, Mean ± SD) |  | 0.3±0.9 | 0.2±0.8 | 0.6±0.9 | 0.3±0.9 | 0.293 |
| Retained Natural Tooth (N, Mean ± SD) | | 27.4±1.4 | 26.5±2.7 | 24.7±2.9 | 26.3±2.6 | 0.001 |
| Sex (n,(%)) |  |  |  |  |  | 0.154 |
|  | Female | 15(%60.0) | 17(%45.9) | 7(%31.8) | 39(%46.4) |  |
|  | Male | 10(%40.0) | 20(%54.1) | 15(%68.2) | 45(%53.6) |  |
| Drinking (n,(%)) |  |  |  |  |  | 0.268 |
|  | No | 6(%25.0) | 12(%32.4) | 10(%47.6) | 28(%34.1) |  |
|  | Yes | 18(%75.0) | 25(%67.6) | 11(%52.4) | 54(%65.9) |  |
| Smoking (n,(%)) |  |  |  |  |  | 0.762 |
|  | Non-Smoker | 20(%90.9) | 33(%89.2) | 19(%95.0) | 72(%91.1) |  |
|  | Smoker | 2(%9.1) | 4(%10.8) | 1(%5.0) | 7(%8.9) |  |
| Hypertension (n,(%)) |  |  |  |  |  | 0.221 |
|  | No | 22(%88.0) | 28(%75.7) | 14(%66.7) | 64(%77.1) |  |
|  | Yes | 3(%12.0) | 9(%24.3) | 7(%33.3) | 19(%22.9) |  |
| Diabetes (n,(%)) |  |  |  |  |  | 0.16 |
|  | No | 23(%92.0) | 34(%91.9) | 16(%76.2) | 73(%88.0) |  |
|  | Yes | 2(%8.0) | 3(%8.1) | 5(%23.8) | 10(%12.0) |  |
