# Supplementary material for: Machine learning reveals microbiome differences by periodontitis severity
Source: PLoS One. 2026 May 21;21(5):e0349686. doi: 10.1371/journal.pone.0349686 (PMC13193413; doi:10.1371/journal.pone.0349686)
Supplement: S2 Table — (DOCX) [file pone.0349686.s002.docx]

S2 Table. Tooth-level clinical measurements and periodontal parameters.

| Variable (Mean ± SD) | Comparison | Mean Difference | t-statistic | P-value (unadjusted) | Bonferroni P-value | Significant |
| --- | --- | --- | --- | --- | --- | --- |
| Missing Teeth | Mild vs Moderate | -0.6 | -1.174 | 0.245 | 0.735 | Not Significant |
| (Mild: 0.6±1.4, Moderate: 1.2±2.6, Severe: 3.0±2.9) | Mild vs Severe | -2.4 | -3.536 | 0.001 | 0.004 | Significant |
|  | Moderate vs Severe | -1.8 | -2.395 | 0.021 | 0.064 | Not Significant |
|  |  |  |  |  |  |  |
| Implants | Mild vs Moderate | -0.4 | -1.305 | 0.199 | 0.598 | Not Significant |
| (Mild: 0.1±0.4, Moderate: 0.5±1.8, Severe: 1.4±2.2) | Mild vs Severe | -1.3 | -2.732 | 0.012 | 0.036 | Significant |
|  | Moderate vs Severe | -0.9 | -1.623 | 0.113 | 0.339 | Not Significant |
|  |  |  |  |  |  |  |
| Retained Natural Teeth | Mild vs Moderate | 0.9 | 1.715 | 0.092 | 0.275 | Not Significant |
| (Mild: 27.4±1.4, Moderate: 26.5±2.7, Severe: 24.7±2.9) |  |  |  |  |  |  |
|  | Mild vs Severe | 2.7 | 3.978 | 0 | 0.001 | Significant |
|  | Moderate vs Severe | 1.8 | 2.365 | 0.023 | 0.068 | Not Significant |
