# Supplementary material for: Machine learning reveals microbiome differences by periodontitis severity
Source: PLoS One. 2026 May 21;21(5):e0349686. doi: 10.1371/journal.pone.0349686 (PMC13193413; doi:10.1371/journal.pone.0349686)
Supplement: S3 Table — (DOCX) [file pone.0349686.s003.docx]

S3 Table. Comparison of Model Performance Across Classification Algorithms

| Model | Sensitivity | Specificity | Accuracy | F1-score | AUC |
| --- | --- | --- | --- | --- | --- |
| SVM | 0.00 | 1.00 | 0.53 | 0.35 | 0.69 |
| KNN | 0.43 | 0.38 | 0.40 | 0.40 | 0.50 |
| DT | 0.71 | 0.75 | 0.73 | 0.73 | 0.71 |
| RF | 0.86 | 1.00 | 0.93 | 0.93 | 0.98 |
| XGBoost | 0.86 | 1.00 | 0.93 | 0.98 | 0.98 |

AUC, area under the curve; SVM, support vector machine; KNN, k-nearest neighbors; DT, decision tree; RF, random forest; XGBoost, extreme gradient boosting
