# Supplementary material for: Machine learning reveals microbiome differences by periodontitis severity
Source: PLoS One. 2026 May 21;21(5):e0349686. doi: 10.1371/journal.pone.0349686 (PMC13193413; doi:10.1371/journal.pone.0349686)
Supplement: S4 Table — (DOCX) [file pone.0349686.s004.docx]

S4 Table. Performance Metrics of XGBoost Model according to populations

| Population | Sensitivity | Specificity | Accuracy | F1-score |
| --- | --- | --- | --- | --- |
| Korean | 0.67 | 0.90 | 0.85 | 0.79 |
| Chinese | 0.92 | 0 | 0.55 | 0.36 |
| Spanish / Portuguese | 0.5 | 0.25 | 0.40 | 0.38 |
