# Supplementary figures and images for: Machine learning reveals microbiome differences by periodontitis severity
Source: PLoS One. 2026 May 21;21(5):e0349686. doi: 10.1371/journal.pone.0349686 (PMC13193413; doi:10.1371/journal.pone.0349686)

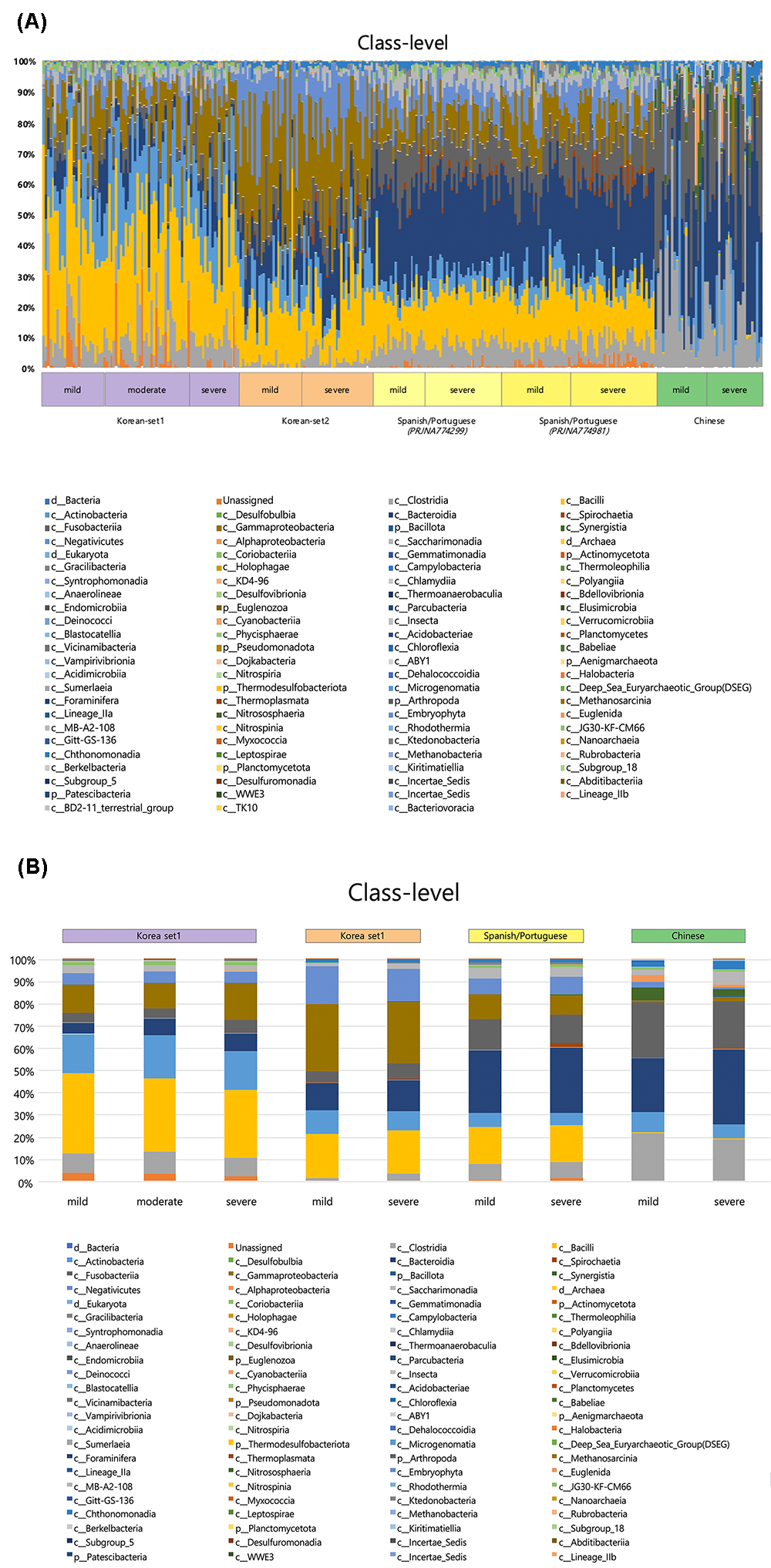

Supplement: S1 Fig — (TIF) [file pone.0349686.s005.tif]
